# Divergence of the SigB regulon and pathogenesis of the *Bacillus cereus sensu lato* group

**DOI:** 10.1186/1471-2164-13-564

**Published:** 2012-10-22

**Authors:** Edgar Scott, David W Dyer

**Affiliations:** 1 Department of Microbiology and Immunology, Oklahoma University Health Sciences Center, 975 NE 10th St., BRC 1106, Oklahoma City, OK, 73117, USA

**Keywords:** Microbial evolution, Generalized stress response, SigB sigma factor

## Abstract

**Background:**

The *Bacillus cereus sensu lato* group currently includes seven species (*B. cereus*, *B. anthracis*, *B. mycoides*, *B. pseudomycoides*, *B. thuringiensis*, *B. weihenstephanensis* and *B. cytotoxicus*) that recent phylogenetic and phylogenomic analyses suggest are likely a single species, despite their varied phenotypes. Although horizontal gene transfer and insertion-deletion events are clearly important for promoting divergence among these genomes, recent studies have demonstrated that a major basis for phenotypic diversity in these organisms may be differential regulation of the highly similar gene content shared by these organisms. To explore this hypothesis, we used an *in silico* approach to evaluate the relationship of pathogenic potential and the divergence of the SigB-dependent general stress response within the *B. cereus sensu lato* group, since SigB has been demonstrated to support pathogenesis in *Bacillus*, *Listeria* and *Staphylococcus* species.

**Results:**

During the divergence of these organisms from a common “SigB-less” ancestor, the placement of SigB promoters at varied locations in the *B. cereus sensu lato* genomes predict alternative structures for the SigB regulon in different organisms. Predicted promoter changes suggesting differential transcriptional control of a common gene pool predominate over evidence of indels or horizontal gene transfer for explaining SigB regulon divergence.

**Conclusions:**

Four lineages of the SigB regulon have arisen that encompass different gene contents and suggest different strategies for supporting pathogenesis. This is consistent with the hypothesis that divergence within the *B. cereus sensu lato* group rests in part on alternative strategies for regulation of a common gene pool.

## Background

The genus *Bacillus* is a heterogeneous group of Gram-positive heterotrophic aerobic or facultative anaerobic bacilli that form environmentally resistant, metabolically inert spores [1]. These soil-borne organisms are ubiquitous, and occupy surprisingly diverse environments [2,3]. Within this large genus, the *B. cereus sensu lato* group consists of seven species *B. anthracis* (*Ba*), *B. cereus* (*Bc*), *B. mycoides*, *B. pseudomycoides*, *B. thuringiensis* (*Bt*), *B. weihenstephanensis* (*Bw*) and *B. cytotoxicus*, based on classical microbial taxonomy [4,5]. More recent molecular phylogenies and comparative genome sequence analysis indicate that these organisms should more accurately be viewed as a single species [6,7] despite their phenotypic diversity. Indeed, the various species of the *Bc sensu lato* group are phylogenetically interspersed among one another in several phylogenies [8-10]. Although the population has a clonal character, there do not appear to be clonal lineages that are species-specific, with the exception of the *Ba* lineage [11]. Some *Bc sensu lato* organisms are thermophiles [12], while *Bw* is a psychrophile [4]. Nevertheless, most *Bc sensu lato* isolates are mesophiles, found in a breadth of locales including the soil, on plant surfaces and the mammalian gastrointestinal microflora [13]. Some *Bc sensu lato* members appear to be nonpathogenic, while others cause diverse disease ranging from food poisoning (intoxication without colonization) to gastroenteritis [13], endophthalmitis [14], tissue abscesses [15,16], and aggressively invasive systemic disease, including anthrax [3]. *Bt* strains can cause disease in insects [17,18] and possibly nematodes [19-21], while some *Bc* strains are part of the normal insect gut flora [13,22].

Thus, these organisms appear to have arisen from a common ancestor to display impressive phenotypic diversity while nevertheless occupying a close phylogenetic space. What mechanisms produced this dichotomy? Horizontal gene transfer (HGT), mobile genetic elements, and the routine processes of insertion/deletion (indel) formation have typically been invoked to explain the diversity in these organisms [23,24]. Clearly, the presence of mobile genetic elements such as the virulence plasmids pXO1 and pXO2 in the *Ba* lineage, and the Cry toxin plasmids in various *Bt* strains, are essential for the signature phenotypes of these organisms [3,25]. On the other hand, it is unclear how much of the phenotypic diversity in these organisms can be explained by these mechanisms. As a whole, *Bc sensu lato* organisms have an extremely high degree of chromosomal synteny [26], and whole genome comparisons between these organisms reveal a highly similar gene content [26,27]. Han *et al.*[27] suggested “that differential regulation [of gene content] modulates virulence rather than simple acquisition of virulence factor genes”, a conclusion confirmed by other studies [28]. This proposition is consistent with observations that the most evolutionarily flexible portions of the bacterial genome are regulatory sequences and transcriptional networks [29-31].

We decided to explore the divergence within the *Bc sensu lato* group by examining the divergence of the SigB regulons in these organisms. The *sigB* locus encodes an alternative sigma factor with orthologs confined to the *Bacillus*, *Staphylococcus* and *Listeria* genera [32]. In each of these, the SigB protein is responsible for transcriptionally activating the generalized stress response when induced by a variety of stressors, including heat, osmolarity, organic solvents, low pH or cell-wall active antibiotics [33-35]. In these organisms, SigB appears to control virulence-related functions including biofilm formation and invasion [36,37]. Some data that suggest that components of the SigB-mediated stress response vary between strains of *Listeria* and of *S. aureus* in a lineage-dependent manner [38-40]. This suggests that part of the evolutionary differentiation that occurred within these genera included divergence of the structure of the SigB-controlled regulons.

The divergence of the SigB regulon in the *Bc sensu lato* group appears to be similar. Lapidus *et al.*[4] observed that *B. cytotoxicus* [formerly *Bc* biovar *cytotoxis*[41]] is likely the most similar of the *Bc sensu lato* organisms to the nearest common ancestor of the group, based on comparative genome analysis and 16S rRNA phylogeny. Our whole genome single-gene phylogeny [7] supports the placement of *B. cytotoxicus* at the base of the phylogeny of the *Bc sensu lato* group. Important in the present context, the *B. cytotoxicus* genome lacks the entire SigB operon [4], including the SigB gene and the primary regulatory loci that control SigB activity, RsbV (anti-sigB antagonist) and RsbW (anti-sigB factor). Consequently, the *B. cytotoxicus* genome does not encode either the sigma factor or associated SigB regulatory genes, and cannot mount a SigB-activated stress response. Lapidus *et al.* suggested that an organism similar to *B. cytotoxicus* was likely the ancestor of the remainder of the *Bc sensu lato* lineage, which arose after receiving the SigB operon during an HGT event. Consistent with this, we were unable to find convincing evidence of SigB binding sites in the *B. cytotoxicus* genome that correspond to SigB promoters in other members of the *Bc sensu lato* group (see below). An alternative explanation, that *B. cytotoxicus* had lost the SigB operon and other genomic information during streamlining of a larger genome [42,43], is less likely. In that instance, we expect that detectable remnants of the SigB regulon (e.g., SigB promoters, pseudogenes) would remain in the *B. cytotoxicus* genome. Thus, the introduction of an intact SigB operon into a *B. cytotoxicus*-like ancestor, likely by HGT as suggested by Lapidus *et al.*, appears to have set the stage for the emergence of the SigB-controlled stress response regulon in the entire *Bc sensu lato* group. From this perspective, the *B. cytotoxicus* genome therefore is a convenient lens through which to view the appearance and divergence of the SigB regulon in the remainder of the *Bc sensu lato* group. Exploiting experimental data derived from *Bc* strain ATCC14579 [44,45], we used an *in silico* approach to predict and compare the SigB regulons of the completed genomes of 20 members of the *Bc sensu lato* group. Not surprisingly, protein coding sequence indels play an important role in the divergence of these regulons. However, changes in promoter sequence between members of the *Bc sensu lato* group that ‘re-purpose’ conserved genes into/out of the SigB regulon appear to be more common than indel formation for remodeling the structure of this regulon during divergence. Four different lineages of the SigB regulon appear to have arisen during this process. One lineage appears to carry the core SigB regulon that arose after the emergence of these organisms from a *B. cytotoxicus*-like ancestor. This lineage appears to have given rise to three additional groups that each appropriated different genes from a common gene pool into the SigB regulon, suggesting different strategies for the support of pathogenesis by the SigB-mediated generalized stress response.

## Results and discussion

### SigB binding site model building and regulon predictions

**Figure 1:**
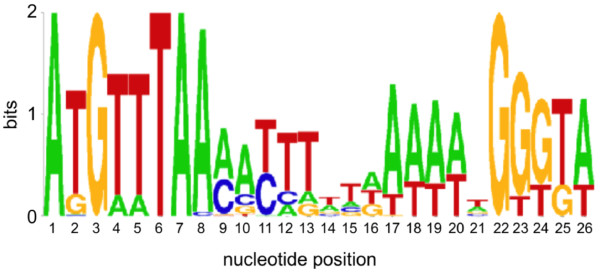
**Seqlogo describing the SigB binding site model derived in this study.** This model is virtually identical to the *Bc* ATCC14579 SigB binding site derived by van Schaik *et al.*[44], except that the A at position 8 has a stronger signal, and the spacer region between the −35 and −10 sequences is shorter by 1bp. Since the *Bc* ATCC14579 binding site was derived from 10 sequences, while our SigB binding site model relied on 130 sequences, our model may be somewhat more robust, although the differences appear negligible. A model of the binding site for the orthologous *B. subtilis* SigB protein also is very similar, lacking only the strong A at position 7; the *B. subtilis* SigB binding site was extracted from a training set of 63 sequences [47].

We began with nine 150bp DNA sequences encompassing the SigB-dependent promoters identified by Van Schaik *et al.*[44], expanded to 166 sequences by phylogenetic footprinting [46]. Redundant sequences were removed from this collection to yield a final training set of 130 sequences. The training set was then used in parallel to develop separate HMM and PWM models for the SigB-dependent promoters in the *Bc sensu lato* group. Our models are virtually identical to previously derived models for this DNA binding site in *Bc* ATCC14579 [44] and *B. subtilis*[47] (Figure 1). Each model was used to scan all genomes for potential SigB binding sites, and this information was coupled with transcriptome-derived transcriptional unit (TU) predictions to arrive at a predicted regulon structure for each organism.

**Figure 2:**
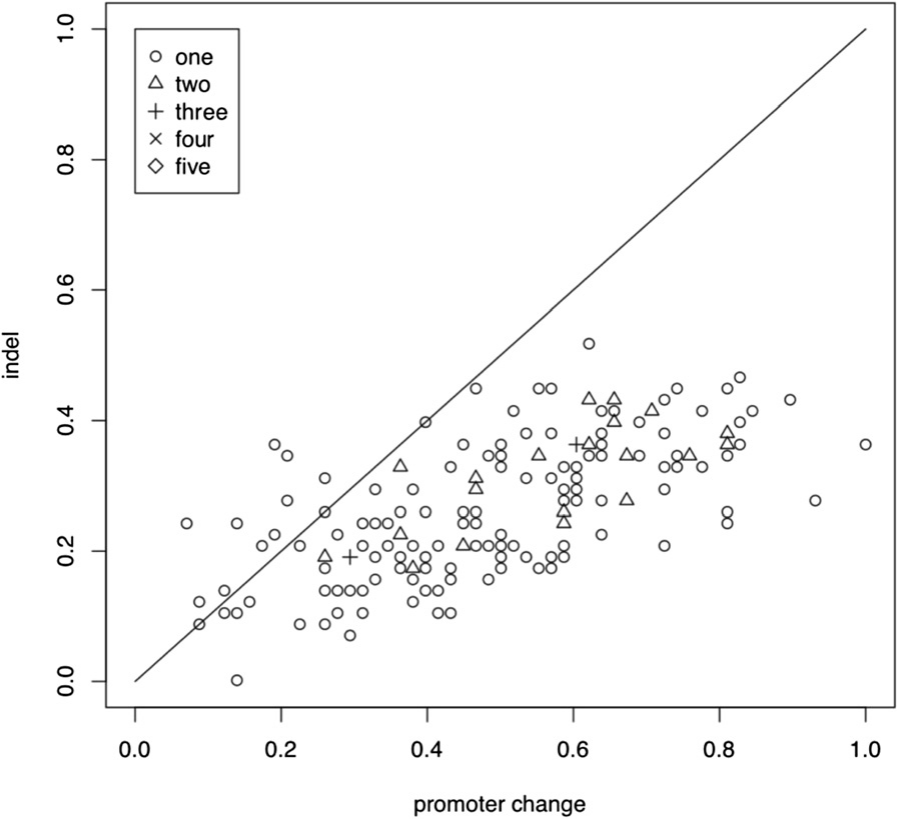
**Pairwise comparisons of the structure of the predicted SigB regulons of the *Bc sensu lato *group.** Comparisons expressed as the ratio of promoter changes vs. indels for each pairwise comparison. If the number of promoter changes and indels for a given pairwise comparison were equivalent, then the value of this ratio would appear on the diagonal line across the center of the plot. Points above this line indicate a pairwise comparison where differences in the SigB regulons relied more commonly on indels rather than promoter changes. Conversely, points below this line denote a ratio arising from pairwise comparison where the differences between SigB regulons revealed a greater number of occurrences of reassignment of common genes into/out of the SigB regulon due to the appearance/disappearance of a predicted SigB promoter. The number of paired genomes yielding each ratio are plotted as one (○), two (), three (**+**), four (**×**) or five (◊) pairs.

**Figure 3:**
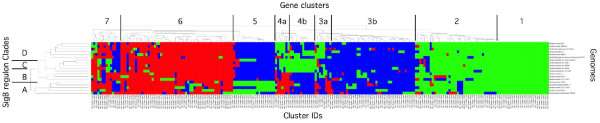
**Heat map comparing the SigB regulons of 19 *Bc sensu lato *genomes.** This heat map is color-coded as: Red, gene absent from the genome; Blue, gene present in the genome, but lacking a SigB promoter binding site 5’ to the beginning of the predicted TU; Green, gene present in the genome and in a TU predicted to be controlled by a SigB promoter. Cluster analysis (tree shown on top of heat map) segregated these genes into 7 clusters, and we operationally divided two of these clusters (3 and 4) into subdivisions (a and b). This was done to emphasize that clusters 3a, 4a and 4b appear to contribute to functional differences between these organisms, while cluster 4b does not (see text for explanation). Cluster analysis also indicated that the genomes included in this figure could be assigned to one of four clades, designated A-D, as noted at the one end of the heat map; at the other end, the identity of each genome used in these analyses is listed. This information is summarized in Table 2. The orthologous cluster ID tags for the genes that are predicted to be in these SigB regulons are displayed across the bottom of the heat map. The genome annotations and cluster ID tags are legible in Additional file 1: Figure S1, and are cataloged in Additional file 2: Table S1.

Horizontal gene transfer leading to insertion of novel genes, gene duplication and divergence, or gene deletion events, are thought to be the most common mechanisms responsible for remodeling bacterial genome function. Collectively, these events can be easily identified as the insertion or deletion (indel) of protein coding sequence, although the underlying mechanism responsible for the appearance of an indel is not so easily surmised. Surprisingly, preliminary manual comparison of the predicted SigB regulons of the *Bc sensu lato* group suggested that the appearance of indels was not the predominant mechanism of divergence of these SigB regulons. In the majority of instances, differences between SigB regulon structure appeared to arise from the placement of SigB-specific promoters in the 5’ region of TUs that were shared among these organisms. That is, SigB regulon divergence appeared to rely on a process of promoter ‘re-assignment’ of genes from a common gene pool into/out of the SigB regulon. This promoter re-assignment appears to predominate over instances of indels that changed the total coding capacity of a given SigB regulon. To get a better quantitative measure of this impression, we performed pairwise comparisons of the predicted SigB regulons of all *Bc sensu lato* genomes (Figure 2), by calculating a simple ratio of the number of predicted promoter changes to the number of observed indel events, for each pair of regulons. A promoter change occurs during the comparison of DNA sequence upstream of orthologous genes in which one gene contains a predicted binding site with a significant score while the second either does not contain a predicted binding site, or contains a predicted binding site that has a score that is not significant. The results confirmed that the predominant mechanism for divergence of these regulons relied on the assignment of new SigB promoters to genes from a common gene pool (Figure 2). We extended this comparison by constructing a heat map summarizing the structures of the predicted SigB regulons in the *Bc sensu lato* group (Figure 3; a larger version of this figure with legible annotation is presented as Additional file 1: Figure S1). This heat map is color-coded: green blocks indicate the presence of a gene predicted to be in a TU controlled by a SigB promoter, while blue blocks indicate the presence of an orthologous gene not controlled by SigB. Red blocks indicate that the ortholog in question is absent from the genome.

Cluster analysis of the genes predicted in the SigB regulons of these genomes grouped these genes into seven clusters; within these, we identified an additional three subclusters that appear to have functional significance (Figure 3, clusters 3a, 4a, and 4b). This cluster analysis defined a core SigB regulon (clusters 1 and 2) that included genes common to the SigB regulon in all *Bc sensu lato* organisms. Additions to this core regulon (clusters 3–7) appear to be clade-specific. It is important to note that the HMM and PWM scores for each of the predicted promoters in clusters 3–7 were statistically indistinguishable from those in clusters 1 and 2. That is, the predictions for the promoters for gene clusters 3–7 were as strongly supported as those in the core SigB regulon, including TUs for which supporting experimental evidence exists [44]. Note also that the bulk of gene cluster 3 (excepting cluster 3a), and clusters 6 and 7 provide relatively modest additions to the core SigB regulons of these organisms. In contrast to gene clusters 1, 2, 3a, 4a/b and 5, there does not appear to be any pattern to the additions in clusters 3b, 6 and 7. Instead, these gene clusters appear to represent noise in the data set. Since the genomes examined in this study were annotated by as many as eight different sequencing groups over a span of seven years (Table 1), the lack of standardization in these various annotation schemes appears to be the basis for much of the noise in these gene clusters (I. Toby and D. Dyer, manuscript in preparation).

**Table 1:** Genomes used in this study

					**Predicted Operons**		
** Organism**^**1**^	**Genbank Accession**	**Annotation date**	**Sequencing group**^**3**^	**Locus tag**	**monocistronic**	**polycistronic**	**total**
Bw KBAB4	NC_010184	05/2010	HAU	KBAB	2106	809	2915
Bc B4264	NC_011725	12/2008	JCVI	BCB	2187	855	3042
Bc ATCC14579	NC_004722	03/2003	INRAGM	BC	2163	840	3003
Bt BMB171	NC_014171	05/2010	HAU	BMB	2135	837	2972
Bc G9842	NC_011772	10/2008	JCVI	BCG	2271	847	3118
Bc Q1	NC_011969	01/2009	MGCC	BCQ	2005	855	2860
Bc AH187	NC_011658	12/2008	JCVI	BCAH187	2199	858	3057
Bc ATCC10987	NC_003909	09/2004	TIGR	BCE	2397	825	3222
Ba Ames Ancestor	NC_007530	03/2003	TIGR	GBAA	2146	841	2987
Ba Ames	NC_003997	03/2003	TIGR	BA	2081	834	2915
Ba A0248	NC_012659	05/2009	LANL	BAA	1995	821	2816
Ba Sterne^2^	NC_005945	01/2004	JGI	BAS	2245	938	3183
Ba CDC 684	NC_012581	04/2009	LANL	BAMEG	2245	861	3106
Bc E33L	NC_006274	11/2004	JGI	BCZK	1930	842	2772
Bc 03BB102	NC_012472	03/2009	LANL	BCA	2135	857	2992
Bt Al-Hakam	NC_008600	11/2006	JGI	BALH	1812	817	2629
Bc CI	NC_014335	07/2010	GGL	BACI	1963	862	2825
Bc AH820	NC_011773	10/2008	JCVI	BCAH820	2168	858	3026
Bt konkukian 97-27	NC_005957	12/2004	JGI	BT	1907	844	2751
Bcy NVH-391-98	NC_009674	07/2007	JGI	Bcer98	1687	589	2276

^1^ Ba, *B. anthracis*, Bc, *B. cereus*, Bt, *B. thuringiensis*, Bw, *B. weihenstephanensis,* Bcy, *B. cytotoxicus.*

^2^ Operon data experimentally determined by Passalacqua *et al.*[28].

^3^JGI: Joint Genome Institute.

LANL: Los Alamos National Labs.

JCVI: J. Craig Venter Institute.

TIGR: The Institute for Genome Research.

INRAGM: INRA Genetique Microbienne.

MGCC: Microbial Genome Center of Chinese Ministry of Public Health.

HAU: Huazhong Agricultural University.

GGL: Goettingen Genomics Laboratory.

### The core SigB regulon

Analysis of the SigB regulons in each genome suggested that these organisms could be assigned to four clades A through D (Table 2), based on the constituents of the SigB regulon predicted for each organism. Clade C consists exclusively of *B. anthracis* strains; this is not surprising, as these organisms are highly monomorphic. The size of this clade in comparison to Clades A, B and D is somewhat misleading, due to the overrepresentation of these genomes in the data set. Nevertheless, this cluster analysis approximates relationships that closely mirror other phylogenetic hypotheses for the *Bc sensu lato* group [8]; this is significant in that the relationships in Figure 3 are based on the structure of the SigB regulons (SigB promoters and SigB-controlled genes), rather than from DNA or protein sequence alone. The core SigB regulon (gene clusters 1 and 2) includes essentially all of the SigB-controlled genes found in Clade B organisms, with few exceptions. Comparison of this core regulon to the *B. cytotoxicus* genome (Table 3; 14 TUs) revealed that 20 of 47 (43%) genes in the core regulon are orthologous to *B. cytotoxicus* genes. Assuming that the *B. cytotoxicus* genome reflects the ancestral state prior to the introduction of SigB into this lineage, this suggests that the genes listed in Table 3 pre-dated the introduction of SigB into the *Bc sensu lato* lineage. These genes were subsequently recruited into the SigB core regulon by the assignment of a SigB promoter after the appearance of SigB. Orthologs of the remaining 23 genes of the SigB core regulon (total of 43 genes) were not found in *B. cytotoxicus*, and presumably were added later. At least 20 of the core regulon genes encode hypothetical proteins, presumably arising in the *Bc sensu lato* group by HGT. Most of the remainder of the genes in this group are members of large gene families (e.g., sensor kinases, permease subunits) that likely became part of the SigB regulon by promoter re-assignment, possibly following a gene duplication event. However, the timing of the introduction of the SigB operon into the *Bc sensu lato* group and the appearance of these genes is uncertain, as is when these genes became incorporated into the SigB regulon.

**Table 2:** SigB regulon clades

**SigB regulon Clade**	**Organism**	**Source or location of isolation**
***Clade A***	*B. weihenstephanensis* KBAB4	Soil
	*B. cereus* B4264	Bloodstream isolate from pneumonia patient
	*B. cereus* ATCC 14579	Dairy product
	*B. thuringiensis* BMB171	Soil
	*B. cereus* G9842	Stool sample from food poisoning outbreak
***Clade B***	*B. cereus* Q1	Deep oil reservoir
	*B. cereus* AH187	Dairy product
	*B. cereus* ATCC 10987	Cheese spoilage
***Clade C***	*B. anthracis* Ames Ancestor	Bovine carcass
	*B. anthracis* Ames	Bovine carcass
	*B. anthracis* A0248	Human disease
	*B. anthracis* Sterne	Vaccine strain
	*B. anthracis* CDC684	NA
***Clade D***	*B. cereus* E33L	Zebra carcass
	*B. cereus* 03BB102	Human blood isolate
	*B. thuringiensis* Al-Hakam	Iraq bioweapons facility
	*B. cereus* Cl	Chimpanzee carcass
	*B. cereus* AH820	Human periodontitis
	*B. thuringiensis* konkukian 97-27	Human tissue necrosis
***No regulon evident***	*B. cytotoxicus* NVH-391-98	Food poisoning outbreak

**Table 3:** ***B. cytotoxicus* genes that pre-date the* Bc sensu lato* core SigB regulon**

** Cluster ID**	**TU**	**Representative locus tag**	**Annotation**
bID_Cluster_3759	1	Bcer98_0620	thiamine/molybdopterin biosynthesis ThiF/MoeB-like protein
bID_Cluster_5471	1	Bcer98_0621	phosphomethylpyrimidine kinase
bID_Cluster_1960	2	Bcer98_2653	PhoH family protein
bID_Cluster_3960	3	Bcer98_3311	N-6 DNA methylase
bID_Cluster_9165	3	Bcer98_3312	redoxin domain-containing protein
bID_Cluster_10657	3	Bcer98_3313	hypothetical protein
bID_Cluster_10524	4	Bcer98_3648	nuclear protein SET
bID_Cluster_8180	5	Bcer98_3694	ATP-dependent Clp protease proteolytic subunit
bID_Cluster_2287	6	Bcer98_3853	transcription termination factor Rho
bID_Cluster_10550	7	Bcer98_0367	methyl-accepting chemotaxis sensory transducer
bID_Cluster_4143	7	Bcer98_0430	NAD-dependent epimerase/ dehydratase
bID_Cluster_1966	8	Bcer98_0498	citrate carrier protein
bID_Cluster_2625	8	Bcer98_0499	malate dehydrogenase, putative
bID_Cluster_3344	9	Bcer98_0651	hypothetical protein
bID_Cluster_2017	10	Bcer98_1017	hypothetical protein
bID_Cluster_6230	11	Bcer98_1200	two component transcriptional regulator, ResD
bID_Cluster_857	11	Bcer98_1201	multi-sensor signal transduction histidine kinase, ResE
bID_Cluster_9721	12	Bcer98_3007	ferric uptake regulator family protein
bID_Cluster_3378	13	Bcer98_3102	hypothetical protein
bID_Cluster_4858	14	Bcer98_4017	MscS mechanosensitive ion channel

All of the SigB-controlled genes originally described by Van Schaik *et al.*[44] are found in this core regulon. We identified 27 additional genes that are predicted to be in the core SigB regulon (Figure 3 and Additional file 2: Table S1). Many of the additional genes encode regulatory proteins, whose altered transcription may be difficult to detect by microarray analysis, and whose regulatory control may be difficult to ascertain in the absence of experimental data. Another large group of genes added to the core regulon are predicted to encode hypothetical proteins. As a consequence, it is difficult in most instances to predict how these additional genes contribute to the function of the core SigB stress response.

The discrepancy between our predictions and the data of Van Schaik *et al.* is not surprising; similar difficulties in cataloguing the complete SigB-dependent response have been encountered in other systems. For instance, as noted by Nannapaneni *et al.*[48], three independent *B. subtilis* studies identified over 100 SigB-dependent target genes, but only 67 genes were commonly identified in all studies. After extensive testing in a variety of experimental conditions including ethanol and butanol shock, osmotic and oxidative stress, low-temperature growth and heat shock, Nannapaneni *et al.* demonstrated 166 genes that appeared to be part of the *B. subtilis* SigB-controlled response; 19 of these also were controlled by secondary regulators. Earlier discrepancies were likely due to differences in growth conditions, microarray platforms and the experimental setups employed. Similarly, the experiments of Van Schaik *et al.* likely identified a subset of the total SigB-dependent genes in *Bc* ATCC14579, as these experiments employed a short heat shock [44] or overexpression of the SigB protein [45], and were not as extensive as those of Nannapaneni *et al.* Secondary regulators interacting with the SigB regulon also are a likely complication. In *S. aureus*, SpoVG fine-tunes SigB-dependent regulation by antagonizing SigB [49]. In *L. monocytogenes*, SigB regulatory control is intertwined with that of HrcA [50], CtsR [51] and PrfA [52], complicating the analysis of SigB-mediated effects. In addition to the secondary regulators found in gene clusters 1 and 2 (Figure 3), additional regulatory proteins are included in clade-specific SigB regulons (see below), and may modulate the control of gene expression by SigB, confounding the results of *in vitro* experiments. Thus, gene clusters 1 and 2 in Figure 3 likely are a more comprehensive representation of the total SigB core regulon in the *Bc sensu lato* group than the early studies of Van Schaik *et al.*.

### Additions to the core SigB regulon

Clade A and Clades C/D organisms appear to have evolved different pathogenic potentials, and this likely is supported by the gene sets added to the core SigB regulon. Clade A organisms have added a group of 20 genes (gene cluster 5, in 4 TUs; Table 4) to the core regulon. The mechanism of addition of this gene cluster to the core SigB regulon almost exclusively rests on the assignment of SigB promoters to TUs found in the common gene pool. This can most easily be seen in Figure 3 (gene cluster 5) as an almost exclusive assignment of either green blocks (genes predicted to be driven by SigB promoters) or blue blocks (orthologous genes present, but lacking a SigB promoter). Only occasionally does one observe an orthologous gene that is absent from a given genome (denoted by a red block in this region of the heat map). Thus, HGT/indels appear to play only a minor role in the assembly of this gene cluster. Clade A includes one member (*Bc* B4264; Table 1) that was isolated from the bloodstream of a pneumonia patient. Although a second Clade A strain, *Bc* ATCC14579, was originally isolated from a dairy product (Table 1), this organism has been shown to cause experimental endophthalmitis following intravitreal injection [53]. This intravitreal injection model mimics the course of disease associated with the entry of the organism into the interior of the eye following traumatic injury. However, this infection model likely by-passes the requirement for direct invasion strategies such as those employed by Clade C/D organisms during anthrax or similarly aggressive disease. The remainder of Clade A includes soil-borne organisms and one organism associated with food-borne intoxication. Thus, Clade A organisms may be pathogenic opportunists, in contrast to Clades C/D organisms, which appear to be frank pathogens (Table 1).

**Table 4:** Gene clusters in Clades A, C and D that augment the SigB core regulon

**Cluster**	**Cluster ID**	**TU**	**Locus tag**	**Annotation**
***Clades C and D***				
** 3a**	bID_Cluster_13222	1	BT9727_2119	spore germination protein PF
	bID_Cluster_527	2	BT9727_2420	FtsI ortholog
	bID_Cluster_845	2	BT9727_2419	sensor histidine kinase
	bID_Cluster_2071	3	BT9727_3346	C4-dicarboxylate transporter DctA
	bID_Cluster_2862	4	BT9727_3495	Hypothetical protein
** 4a**	bID_Cluster_8996	1	GBAA_0583	acetyltransferase
	bID_Cluster_1542	1	GBAA_0584	sensor histidine kinase
	bID_Cluster_6986	1	GBAA_0585	DNA-binding response regulator
	bID_Cluster_4984	2	GBAA_1077	Hypothetical protein
	bID_Cluster_1988	3	GBAA_5500	phosphoglycerate transporter family protein
	bID_Cluster_3664	3	GBAA_5501	putative lipoprotein
	bID_Cluster_5866	3	GBAA_5502	Hypothetical protein
	bID_Cluster_1603	3	GBAA_5503	sensor histidine kinase
	bID_Cluster_6528	3	GBAA_5504	DNA-binding response regulator
	bID_Cluster_3959	3	GBAA_5505	UDP-glucose 4-epimerase
	bID_Cluster_4585	3	GBAA_5506	membrane-bound transcriptional regulator LytR
	bID_Cluster_1186	4	GBAA_5678	ABC transporter ATP-binding protein
** 4b**	bID_Cluster_10476	1	GBAA_1939	Hypothetical protein
	bID_Cluster_5559	2	GBAA_2162	Hypothetical protein
	bID_Cluster_11158	3	GBAA_2384	Hypothetical protein
	bID_Cluster_13854	4	GBAA_2523	HTH DNA-binding protein
	bID_Cluster_618	5	GBAA_3291	Methyl-accepting chemotaxis protein
	bID_Cluster_3242	6	GBAA_3338	S-layer protein
	bID_Cluster_2077	7	GBAA_5674	Hypothetical protein
*** Clade A***				
** 5**	bID_Cluster_6809	1	BMB171_C1598	two-component response regulator, LuxR family
	bID_Cluster_3224	1	BMB171_C1599	sensory transduction protein kinase
	bID_Cluster_4364	1	BMB171_C1600	ABC transporter ATP-binding protein
	bID_Cluster_3195	1	BMB171_C1601	ABC transporter permease
	bID_Cluster_2768	1	BMB171_C1602	ABC transporter permease
	bID_Cluster_2336	1	BMB171_C1603	cardiolipin synthetase
	bID_Cluster_5196	2	BMB171_C2914	Hypothetical protein
	bID_Cluster_3683	3	BMB171_C3448	RecA recombinase
	bID_Cluster_2435	3	BMB171_C3449	Competence damage-inducible protein A
	bID_Cluster_8205	3	BMB171_C3450	Phosphatidylglycerophosphate synthase
	bID_Cluster_4493	3	BMB171_C3451	Hypothetical protein
	bID_Cluster_5830	3	BMB171_C3452	Hypothetical protein, ACT-binding domain
	bID_Cluster_12831	3	BMB171_C3453	Hypothetical protein
	bID_Cluster_6635	3	BMB171_C3454	3-ketoacyl-(acyl-carrier-protein) reductase
	bID_Cluster_2214	3	BMB171_C3455	Predicted Zn-dependent peptidases
	bID_Cluster_2266	3	BMB171_C3456	Predicted Zn-dependent peptidases
	bID_Cluster_3669	3	BMB171_C3457	ABC-type transporter, permease component
	bID_Cluster_3507	3	BMB171_C3458	ABC-type transporter, permease component
	bID_Cluster_1276	3	BMB171_C3459	ABC-type transporter, ATPase component
	bID_Cluster_9779	4	BMB171_C3659	Hypothetical protein

Although Clades C/D genomes encode cluster 5 genes, they are not functionally included in the SigB generalized stress response. Instead, Clades C/D have augmented the core SigB regulon with 24 genes from three different clusters (3a, 4a/b, in 15 TUs; Table 4). Similar to gene cluster 5 in Clade A, gene clusters 3a and 4a have been added to the core SigB regulon primarily by SigB promoter assignment from a common gene pool. By contrast, gene cluster 4b was added to the SigB regulon in Clades C/D by a mixture of HGT/indels and promoter assignment (Figure 3); most of these genes are not present in Clades A and B (red blocks in Figure 3, cluster 4b).

### Clade A and Cluster 5

Cluster 5 includes a two-component regulator not orthologous to those included in Clusters 3a/4a. This suggests that Cluster 5 adds to the core regulon a regulatory cascade that extends further into the *Bc sensu lato* transcriptome, and specific to Clade A. This extension of the core SigB regulon would allow the stress response to be coordinated with the environmental signal(s) to which this secondary regulator independently responds. Gene cluster 5 also appears to coordinate the enhanced synthesis of cardiolipin (CL), stimulated by at least three genes in this cluster, including CL synthase (TU #1), and phosphatidylglycerophosphate synthase and 3-ketoacyl-(acyl-carrier-protein) reductase (TU #2). Increased CL synthesis would change the composition of the cell membrane, increasing the hydrophobicity and viscosity of this membrane [54], which could have varied phenotypic effects. Increased CL levels stimulate protein translocation across the cell membrane in *B. subtilis*[55] and *E. coli*[56], and are important for high osmolarity survival in *B. subtilis*[57] and *S. aureus*[58]. High CL concentrations decrease cell envelope permeability, affecting nonspecific antibiotic resistance in *E. coli*[59], resistance to organic solvents [60], daptomycin resistance in *S. aureus*[61] and *Enterococcus faecalis*[62], and resistance to antimicrobial peptides such as enterococcal AS-48 [63] and platelet microbicidal peptide tPMP-1 [64]. CL-rich domains in *B. subtilis* are preferentially distributed at the medial septa and the poles during exponential growth, and the polar septal membrane and the engulfment and forespore membranes during sporulation [65]. *B. subtilis* spore membranes have a significantly higher CL content than membranes of exponentially growing cells, and CL appears to be essential to the proper functioning of germinant receptors [65].

Due to the extensive divergence within ABC transporter gene family, functional predictions may be difficult to substantiate by sequence similarity alone. Nevertheless, the ABC transporter genes included in TU#1 and TU#3 appear to be most similar to the ABC A superfamily [66], typically responsible for export of hydrophobic compounds. Prokaryotic members of this large gene family include the *Streptomyces peucetius*[67] DrrAB exporter for hydrophobic compounds such as daunomycin and doxorubicin, and the *B. licheniformis* BcrABC bacitracin-resistance proteins [68]. Consequently, the ABC transporters in Cluster 5 may be efflux systems either responsible for CL export or working in concert with enhanced CL production to increase resistance to deleterious hydrophobic compounds or antimicrobial peptides.

Cluster 5 TU #3 also includes the genes encoding RecA and CinA. In *S. pneumoniae*, the CinA ortholog appears to direct RecA to the membrane and enhance competency [69], although in *B. subtilis*, CinA appears to be a nucleoid-associated protein [70]. However, the most obvious consequence of enhanced RecA expression is an increased likelihood of activation of the SOS stress response by cleavage of LexA. In *B. subtilis*, at least 33 genes in 18 TUs appear to be directly under RecA/LexA control [71]. If the *Bc sensu lato* SOS response is similar, this also would extend the SigB generalized stress response into a region of the Clade A transcriptome to make these organisms more apt to express, among others, components of the excision, recombinational and error-prone repair pathways for dealing with DNA damage. Consequently, the collective inclusion of the genes in cluster 5 in the SigB-mediated stress response may fine-tune the stress response for competition with other microorganisms in a mixed microbial milieu. Cluster 5 genes also may confer some advantage during pathogenesis, for example by increasing resistance to the bactericidal effects of neutrophil-induced DNA damage or antimicrobial peptides.

### Clades C/D and Clusters 3a, 4a and 4b

These clusters include the genes for three signal transduction histidine kinases, two of which are in TUs with a (probable) cognate response regulator (Table 4). Two additional transcriptional regulators and a methyl-accepting chemotaxis protein also are included in these gene clusters. This suggests that an entirely different regulatory effector gene cascade extends the core SigB regulon in Clade C/D organisms, presumably coordinating the SigB generalized stress response with different environmental signals than that of Clade A organisms. Note that the two Cluster 4b regulatory proteins are unique to the genomes of invasive Clade C/D organisms, suggesting that these genes likely control functions unique to the invasive phenotype. By contrast, the SigB-responsive regulators in Clusters 3a/4a are part of the common gene pool shared with all genomes, although not included in the SigB regulon in those other organisms. This suggests that regulators found in Clusters 3a/4a are important for supervising aspects of the common metabolism shared with other *Bc sensu lato* organisms, but their inclusion in the generalized stress response is uniquely important for Clades C/D organisms.

Together, these gene clusters allow SigB to coordinate the expression of functions devoted to cell wall and spore structure and biosynthesis. Cluster 3a encodes an FtsI ortholog, also known as PBP-2B [72], which appears to be recruited late to the division septum in *B. subtilis*[73]. One of the cluster 4a regulatory proteins is the ortholog of the *Bacillus* LytR protein, a member of the LytR-CpsA-Psr gene family found in most members of the Firmicutes [74]. In *B. subtilis*, LytR lies in an operon with and controls expression of genes encoding the major autolytic amidase LytC, and a modifier gene LytB [75]. In *Streptococcus pneumoniae*, LytR is essential for normal septum formation [76], while the *S. mutans* LytR controls autolytic activity [77]. The *Staphylococcus aureus* LytR ortholog MsrR plays a role in cell envelope maintenance, cell separation and virulence, and appears to be connected to the *sarA* attenuation and virulence regulatory network [78,79]. Thus, inclusion of LytR in the Clades C/D SigB regulons suggests that controlling cell wall-specific functions is important for supporting invasive disease.

Cluster 4b includes an S-layer protein with three SLH domains, previously designated as BslI by Kern and Schneewind [80]. While no function has yet been ascribed to BslI, the genome of *Ba* Sterne encodes a suite of at least 24 S-layer proteins. Eight other S-layer proteins are involved in peptidoglycan metabolism, with an additional seven important for virulence [80], suggesting that the function of the cell-surface S-layer protein BslI is similar.

Cluster 4a also encodes UDP-glucose 4-epimerase, which catalyzes the conversion of UDP-glucose to UDP-galactose [81]. Transcriptionally linked to this gene by SigB, Cluster 3a includes a member of the GNAT-transacetylase superfamily [82]. Some members of this superfamily use phosphoglucosamine as a substrate for transacetylation [82]. Collectively, these enzymes could collaborate to promote a burst in the synthesis of galactose-N-acetylglucosamine (GalNAc) during the stress response; this disaccharide is an important component of secondary cell wall polysaccharides found in *Ba* and some virulent *Bc* strains [83]. Additionally, GalNAc is an important linker molecule for tethering oligosaccharides to the collagen-like exosporium glycoprotein BclA [81], an immunodominant *Ba* spore antigen [84].

Cluster 3a encodes the gene for spore germination protein GerPF. In *Ba* spores, impairment of GerPF expression causes a germination defect in response to nutrient germinants [85]. This suggests that the SigB stress response in Clades C/D organisms has been adjusted to ensure that a sufficient amount of GerPF is made for proper spore germination potential. Targeting expression of GerPF via SigB does not appear necessary for Clades A/B organisms; possibly GerPF is more important for the proper functioning of spores produced by invasive pathogens. For instance, this protein could be important for proper germination of spores within macrophages, an event that is associated with the ability of *Ba* strains to cause disease [86]. Note also that some cluster 4a genes, while present in Clade D organisms, are not included in the SigB regulon (red blocks in the heat map in the cluster 4a region). This may suggest that the generalized stress response in Clade D microbes is somewhat less capable of augmenting the pathogenic potential of these organisms, in contrast to Clade C organisms, which include only virulent *Ba*.

## Conclusions

### Evolution of the SigB regulon and pathogenesis of the *Bc sensu lato* group

**Figure 4:**
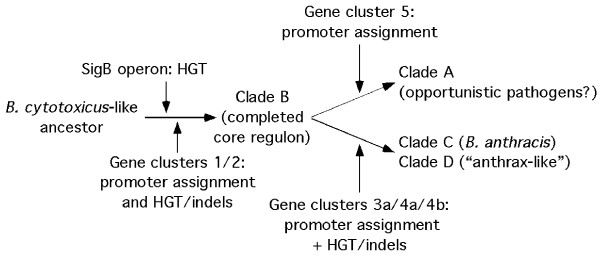
**Proposed pathway for the divergence of the SigB regulons within the *Bc sensu lato *group**.

This sample size is admittedly small, and so the generalizations leading to these hypotheses require further exploration. Nevertheless, the hypotheses drawn from this heat map and cluster analyses are provocative. The simplest interpretation of the accumulated data (Figure 4) suggests that a Clade B-like organism arose first from a *B. cytotoxicus*-like progenitor after receiving the SigB operon during an HGT event. Following this, a nascent core SigB regulon was assembled by the addition of SigB promoters to TUs from the *B. cytotoxicus*-like progenitor. Other genes were added to the core regulon by a combination of HGT and gene duplication/divergence, both events accompanied by SigB promoter assignment. Clade A and Clades C/D organisms subsequently diverged from this common Clade B-like organism by the addition of gene clusters 5 and 3a/4a/b, respectively, to the SigB core regulon. These additions occurred primarily by the assignment of SigB promoters to genes from a common gene pool, while the appearance of new genes, presumably by HGT or gene duplication, appears to be a much less common event. The TUs augmenting the SigB regulon of Clade A organisms may enhance the basal pathogenic potential of this clade, but may also make these organisms more capable of competing in the soil or other microbial consortia. Clades C/D organisms are invasive pathogens; we hypothesize that the augmentation to the SigB core regulon in these organisms is important for supporting this phenotype. Note also that some of the Clade D organisms have been referred to as ‘anthrax-like’ bacteria [27,87], despite the tight clonality of the *Ba* phylogeny that excludes these organisms. The close similarity of the SigB regulon of Clade C and D organisms suggests a functional relationship between these two groups that may be important for their similar pathogenic potential.

## Methods

### Genome sequences used in this study

The completed genome sequences of 20 members of the *B. cereus sensu lato* group used in these studies are listed in Table 1. In this study, we made the simplifying assumption to focus exclusively on whole genome sequences and excluded the extensive collection of draft sequences available for the *Bc sensu lato* group. This was due to our intent to compare the frequency of indels to promoter differences in the SigB regulons of these organisms. Lack of a particular protein coding sequence in a draft genome could be due either to deletion of this coding sequence from the genome, or a lack of sampling of this region during the sequencing for the draft assembly. A related concern was that many draft sequences likely contain regions of misassembly or low sequence quality that could complicate the analyses that we intended, since searching for specific promoter sequences in the genome would require high quality data at all points in the sequence.

### Sigma factor B comparisons

To confirm the identity of the annotated SigB orthologs in each genome, all sigma factor proteins in this genome set were identified using blastp, with the *Bc* ATCC14579 SigB (locus tag BC1004) as the query sequence. These sequences were compared with a maximum likelihood algorithm using PhyML [Seaview [88]] with 100 bootstrap iterations and then analyzed manually to confirm the identity of all BC1004 orthologs. We further examined these proteins to confirm that the DNA binding sites encoded in each protein were the same, and were distinct from those of other sigma factors. The DNA binding sites of all sigma factor proteins encoded in the *Bc* ATCC14579 genome were compared using Sigma70_r2.hmm and Sigma70_r4.hmm (hidden Markov models of sigma factor region 2 and region 4, respectively), obtained from the Pfam website [89]. The utility hmmalign [hmmer-3.0b3 [90]] was used to create alignments of region 2 and region 4 of all *Bc* ATCC14579 primary alternative sigmas, which were manually inspected. A similar comparison was made with all SigB sequences using the utility hmmalign along with Sigma70_r2.hmm and Sigma70_r2.hmm. These comparisons confirmed that all SigB orthologs encoded in these genomes had a conserved DNA binding domain, and therefore are expected to recognize a conserved DNA binding site.

### SigB binding site model building and validation

We began model building by using experimentally-derived SigB binding sites derived from *Bc* ATCC14579 [44,45]. This data set was expanded for training purposes using phylogenetic footprinting [91]. To do this, we began with the 5’ gene of each *Bc* ATCC14579 operon regulated by SigB, including BC0863, BC0998, BC1001, BC1002, BC1005, BC1009, BC1010, BC3132, and BC4641. Blastp [92] was used with default parameters, except an e-value cut-off of 1e-5, to identify similar sequences in the remaining 18 *Bc sensu lato* genomes. We did not use the *B. cytotoxicus* genome in these model-building experiments, as this organism lacks a SigB operon [4]. Orthologs to the *Bc* ATCC14579 proteins in each organism were identified by constructing gene phylogenies for each gene family using Maximum Likelihood analysis (Seaview) and 100 bootstrap iterations. Tblastn searches were performed to ensure that any missing protein sequences in this data set were due to deletion events in the genome in question, rather than being missed during annotation. Then, the 150bp DNA sequence 5’ to each ortholog were collected and analyzed using the MEME default parameters [93] to identify the SigB binding motif 5’ to each gene. These sequences defined the initial training set used for model building. Redundancy in this training was reduced using the purge function from the MEME package. We built two complementary models of the SigB binding site to be used in parallel for all subsequent analyses. A hidden Markov model (HMM) of the training sequences was created using hmmer-1.8.5 [94], while a position weight matrix (PWM) was derived using MEME. Leave-one-out cross validation [95] was used to determine an appropriate empirical *p*-value cut-off of .00273 for the HMM and of 4.61e-7 for the PWM. Background scores for empirical *p*-value calculations for the HMM analysis were determined by searching the SigB HMM against the randomly shuffled *Bc* ATCC14579 genome [shuffleseq from EMBOSS-6.3.1 [96]]. Empirical *p*-values for the SigB HMM alignments were determined by comparing the alignment score to the background scores using a Perl script and R-2.13.0.

### Orthologous Gene Clustering, Operon and Regulon predictions

**Figure 5:**
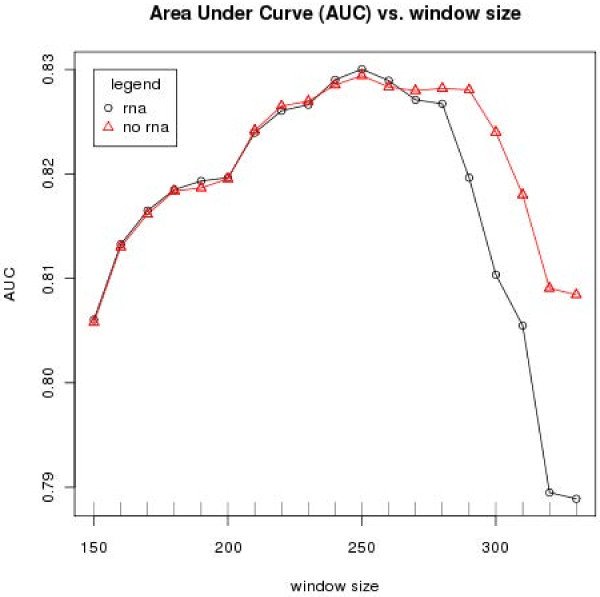
**Receiver-operator characteristic analysis of the predicted *Bc sensu lato *transcriptome.** Plot of the area under the curve (auc) versus the window size parameter of operonMBP for two different datasets. One data set contained RNA annotations while the RNA annotations were not in the second annotation.

Initially, the *Ba* str*.* Sterne transcriptome structure was extracted from the supplementary information provided by Passalacqua *et al.*[28]. For comparing the predicted transcriptomes from all genomes, we clustered all annotated protein-coding sequences using CD-hit [97] to identify orthologous gene clusters. This resulted in a total of 16,835 gene clusters, with cluster sizes ranging from 1 to 19 genes; each cluster was assigned a unique identifier (bID_Cluster_number; see Additional file 3: Table S2). All predicted SigB-regulated genes were combined into a separate file and also clustered with CD-hit. On both data sets, we used the CD-hit default parameters except requiring an 85% sequence identity and an 80% sequence length difference cut-off. Operon predictions were performed using operonMBP [98]. Receiver operator characteristic analysis [99] was applied to the *Ba* str*. Sterne* transcriptome data to determine the appropriate cut-off to maximize true positive and minimize false positive transcription unit (TU) predictions (Figure 5). The appropriate window size was determined to be 250, and the appropriate cut-off for pairing two consecutive genes as co-operonic was 14. All TU predictions were then stored in a MySQL database (see Additional file 4: Table S3 for a summary of the TU predictions for each genome). To predict SigB regulon structure in each organism, the SigB HMM and PWM were used to independently scan across all 20 *Bacillus* genomes, and TUs 3’ to a significant binding site were extracted. We defined significant SigB binding sites as DNA regions that aligned to both the SigB HMM and PWM models, with *p*-values equal to or below at least one of their respective *p*-value cut-offs. Further, we allowed a 5’ untranslated region of up to 4.2 kb, based on the observations of Nicholas *et al.* in *B. subtilis*[100]. This information was then added to the MySQL database.

Finally, the regulon predictions were tabulated in a table of genomes vs. orthologous gene clusters and manually edited for accuracy. Scores of 0, 1, or 2 were assigned to the following states: 0, a gene not present in an orthologous cluster for a specific organism; 1, gene present but not predicted to be regulated by SigB in an orthologous cluster for a specific organism; and 2, gene present and predicted to be regulated by SigB in an orthologous cluster for a specific organism. Then, two comparisons were performed. We first used a pairwise comparison to assess the contribution of SigB promoters and indels to the structure of each regulon, and the changes between genomes. A promoter change occurs during the comparison of DNA sequence upstream of orthologous genes in which one gene contains a predicted binding site with a significant score while the second either does not contain a predicted binding site, or contains a predicted binding site that has a score that is not significant. And, we defined indels simply as the presence or lack of a protein coding sequence at a specific location; other more complex alterations, such as deletion of untranslated regions, SNPs or inversions, were not tabulated. All pairwise regulon comparisons were made in which the number of changes from 2 to 1, or 2 to 0 were counted. These numbers were normalized to the largest value counted and then plotted as indels vs. promoter changes (Figure 2). Secondly, the regulon scores were placed in a table that was converted to a phylip file and analyzed using PHYLIP with Maximum Parsimony and 100 bootstrap iterations to create a SigB regulon phylogeny. This table was also analyzed in R to create a heatmap (Figure 3 and Additional file 1: Figure S1) using heatmap.2 from the library package gplots. Cluster analysis for this heatmap was performed using the default hclust function in R, which specifies a complete-linkage clustering method similar to neighbor-joining.

## Abbreviations

*Ba*: *Bacillus anthracis*; *Bc*: *Bacillus cereus*; *Bw*: *Bacillus weihenstephanensis*; HGT: Horizontal gene transfer; TU: Transcriptional unit; HMM: Hidden Markov Model; PWM: Position Weight Matrix; CL: Cardiolipin; PBP-2B: Penicillin binding protein 2B; SLH: S-layer homology.

## Competing interests

The authors declare that they have no competing interests.

## Authors' contributions

ES and DD designed the studies described in this manuscript. ES performed the initial analyses and ES and DD analyzed the data and co-wrote the paper. All authors read and approved the final manuscript.

## Supplementary Material

Additional file 1 **Figure S1.** Heat map of predicted SigB regulons of 19 *Bc sensu lato* genomes. This heat map is color-coded: green blocks indicate the presence of a gene that is predicted to be in a TU controlled by a SigB promoter, while blue blocks indicate the presence of an orthologous gene that is not controlled by SigB. Red blocks indicate that the ortholog in question is absent from the genome. Click here for file

Additional file 2 **Table S1.** Cluster IDs found in each Gene Cluster identified in Figure 3 and Additional file 1: Figure S1. Cluster IDs generated by CD-hit analysis. Click here for file

Additional file 3 **Table S2.** Cluster IDs for all genes in this study. Click here for file

Additional file 4 **Table S3.** Promoters and TU predictions from this study, for each genome. Click here for file
