# Supplementary material for: Divergence of the SigB regulon and pathogenesis of the Bacillus cereus sensu lato group
Source: BMC Genomics. 2012 Oct 22;13:564. doi: 10.1186/1471-2164-13-564 (PMC3485630; doi:10.1186/1471-2164-13-564)
Supplement: Additional file 1 — Figure S1. Heat map of predicted SigB regulons of 19 Bc sensu lato genomes. This heat map is color-coded: green blocks indicate the presence of a gene that is predicted to be in a TU controlled by a SigB promoter, while blue blocks indicate the presence of an orthologous gene that is not controlled by SigB. Red blocks indicate that the ortholog in question is absent from the genome. [file 1471-2164-13-564-S1.pdf]

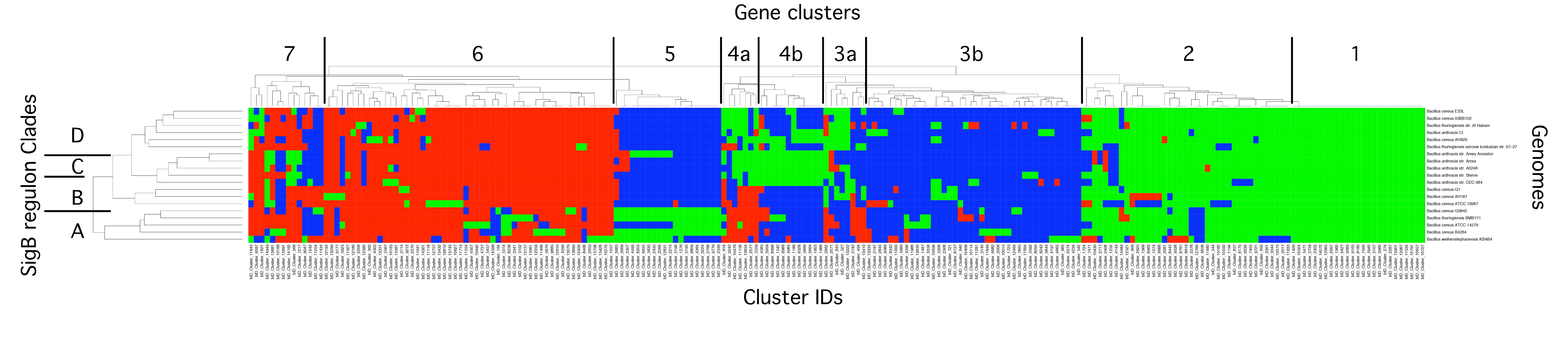

Gene clusters
7
6
5
4a
4b
3a
3b
2
1
SigB regulon Clades
D
C
B
A
Genomes
Cluster IDs
Bacillus cereus E33L
Bacillus cereus 03BB102
Bacillus thuringiensis str. Al Hakam
Bacillus anthracis CI
Bacillus cereus AH820
Bacillus thuringiensis serovar konkukian str. 97-27
Bacillus anthracis str. Ames Ancestor
Bacillus anthracis str. Ames
Bacillus anthracis str. A0248
Bacillus anthracis str. Sterne
Bacillus anthracis str. CDC 684
Bacillus cereus Q1
Bacillus cereus AH187
Bacillus cereus ATCC 10987
Bacillus cereus G9842
Bacillus thuringiensis BMB171
Bacillus cereus ATCC 14579
Bacillus cereus B4264
Bacillus weihenstephanensis KBAB4
